# Supplementary material for: Repurposing Based Identification of Novel Inhibitors against MmpS5-MmpL5 Efflux Pump of Mycobacterium smegmatis: A Combined In Silico and In Vitro Study
Source: Biomedicines. 2022 Jan 31;10(2):333. doi: 10.3390/biomedicines10020333 (PMC8869396; doi:10.3390/biomedicines10020333)
Supplement: Supplementary file 1 [file biomedicines-10-00333-s001.zip › Supplmentary Figures.pptx]

## Slide 1
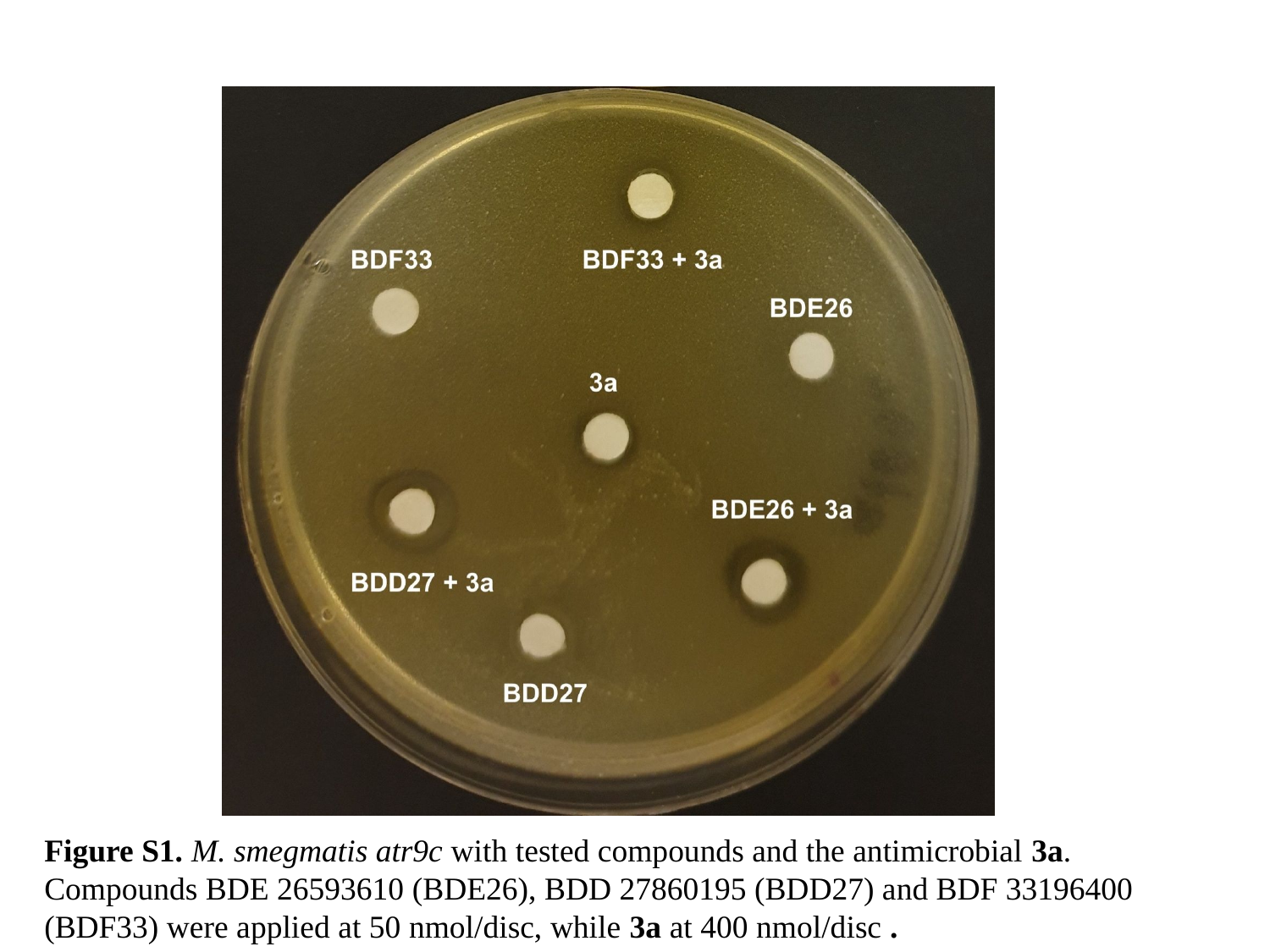

Figure S1. M. smegmatis atr9c with tested compounds and the antimicrobial 3a. Compounds BDE 26593610 (BDE26), BDD 27860195 (BDD27) and BDF 33196400 (BDF33) were applied at 50 nmol/disc, while 3a at 400 nmol/disc .

## Slide 2
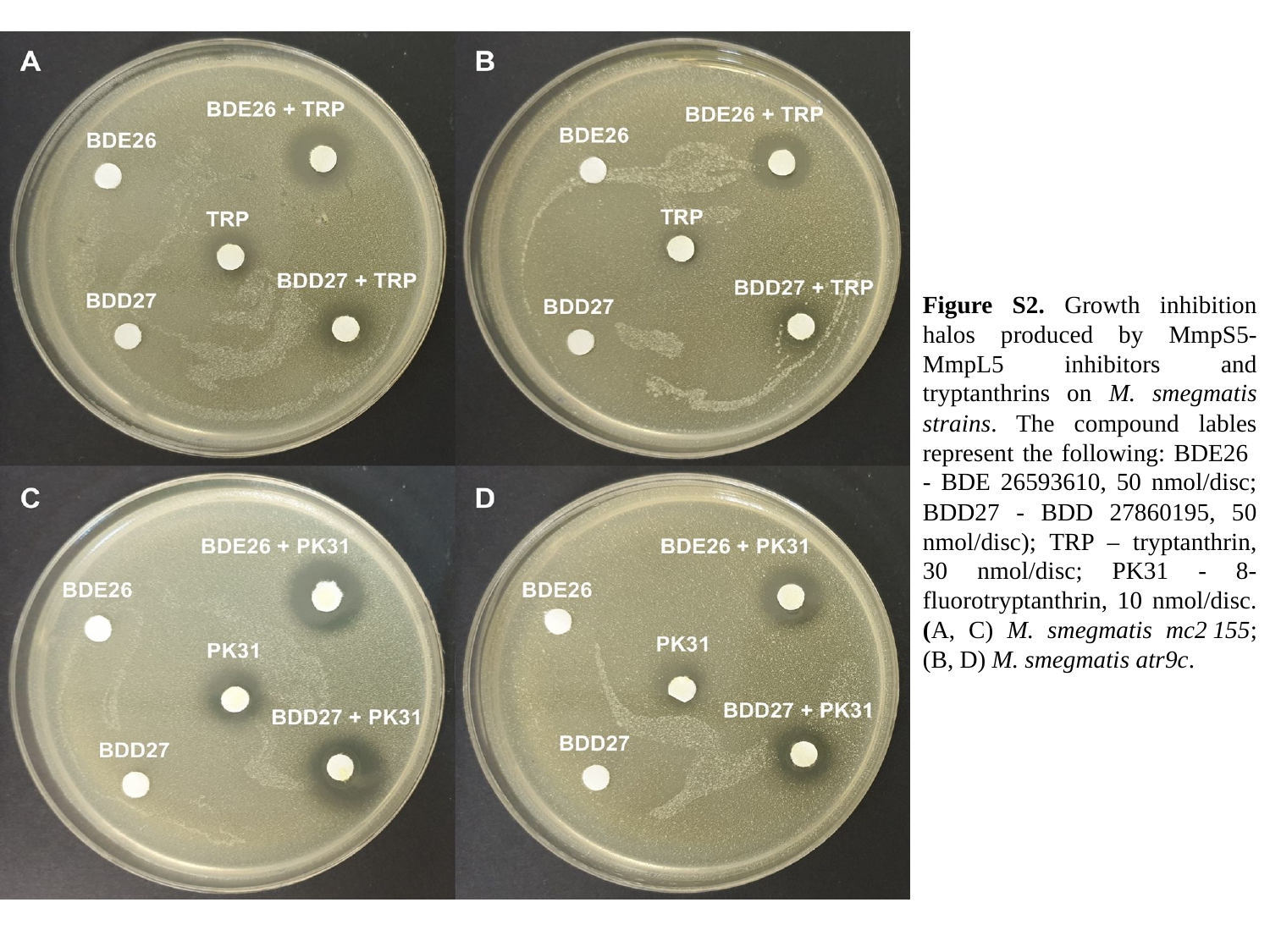

Figure S2. Growth inhibition halos produced by MmpS5-MmpL5 inhibitors and tryptanthrins on M. smegmatis strains. The compound lables represent the following: BDE26 - BDE 26593610, 50 nmol/disc; BDD27 - BDD 27860195, 50 nmol/disc); TRP – tryptanthrin, 30 nmol/disc; PK31 - 8-fluorotryptanthrin, 10 nmol/disc. (A, C) M. smegmatis mc2 155; (B, D) M. smegmatis atr9c.
