# Supplementary material for: Repurposing Based Identification of Novel Inhibitors against MmpS5-MmpL5 Efflux Pump of Mycobacterium smegmatis: A Combined In Silico and In Vitro Study
Source: Biomedicines. 2022 Jan 31;10(2):333. doi: 10.3390/biomedicines10020333 (PMC8869396; doi:10.3390/biomedicines10020333)
Supplement: Supplementary file 1 [file biomedicines-10-00333-s001.zip › Table S1.pdf]

Table S1: The list of compounds collected from the literature for the construction of QSAR models.

S. No	Structure	Name	Molecular Weight (g/mol)	MIC (µg/ml)	pMIC
1.	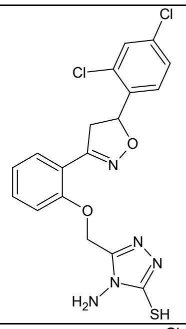	PDS121	436.315	2	5.338
2.	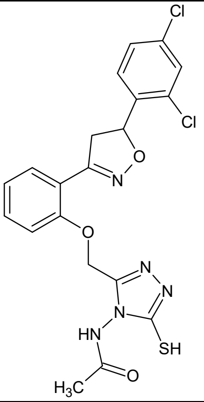	PDS136	478.351	8	4.776
3.	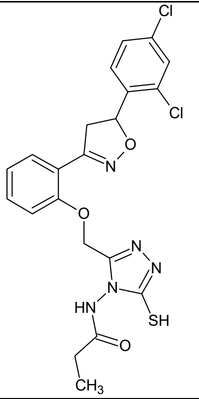	PDS137	492.378	32	4.187

4.	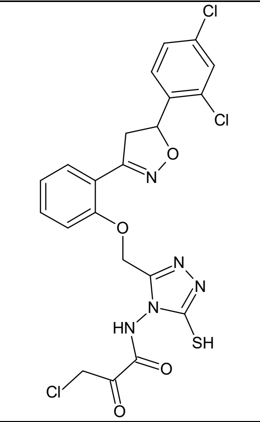	PDS138	540.806	16	4.528
5.	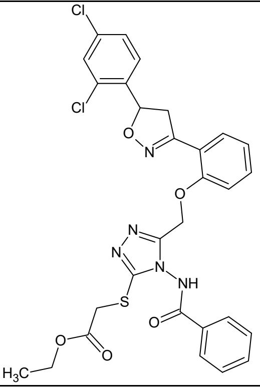	PDS140	626.510	64	3.990
6.	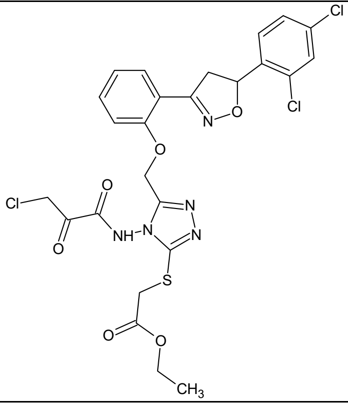	PDS141	626.896	128	3.689
7.	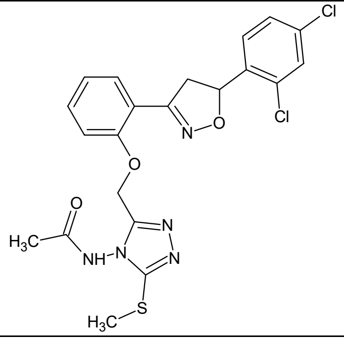	PDS142	492.378	32	4.187

8.	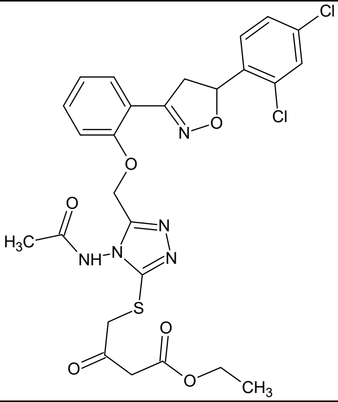	PDS143	606.477	512	3.073
9.	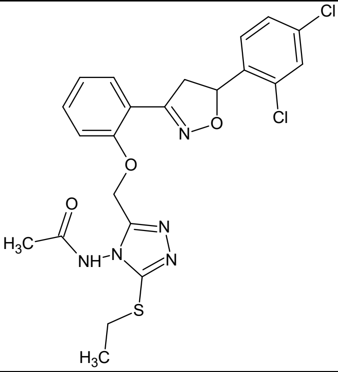	PDS144	506.404	2	5.403
10.	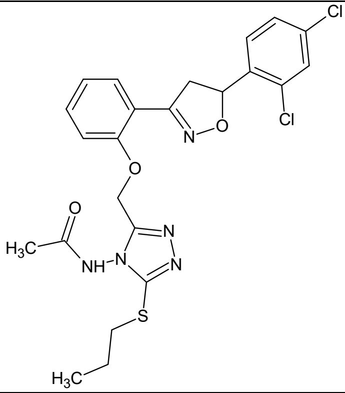	PDS145	520.431	1	5.716
11.	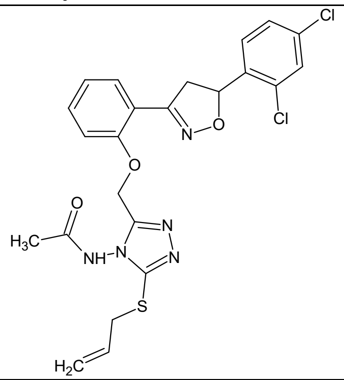	PDS146	518.415	256	3.306

12.	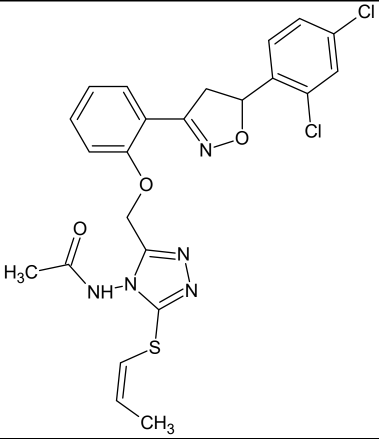	PDS147	518.415	512	3.005
13.	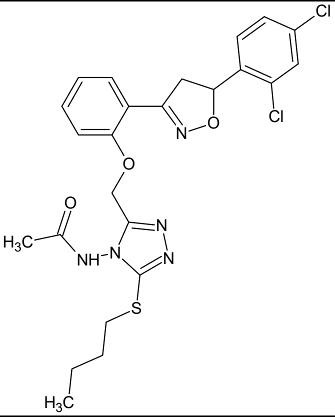	PDS149	534.458	32	4.222
14.	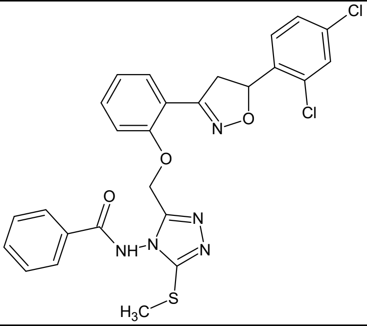	PDS151	554.447	32	4.238
15.	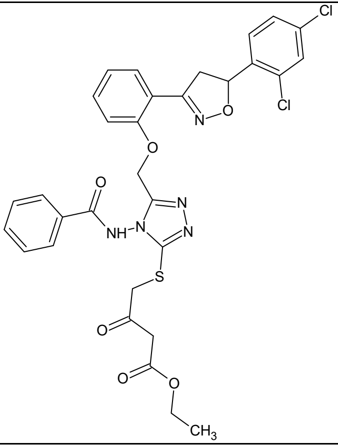	PDS152	668.54698	512	3.115

16.	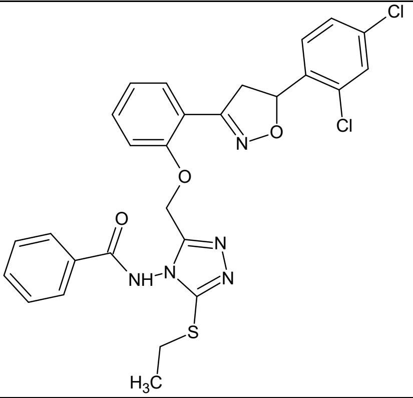	PDS153	568.474	4	5.152
17.	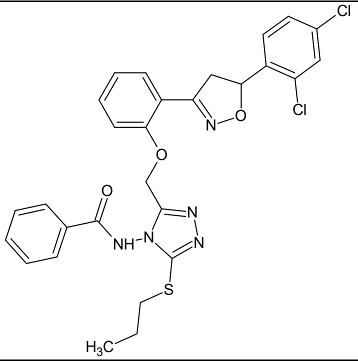	PDS154	582.500	2	5.464
18.	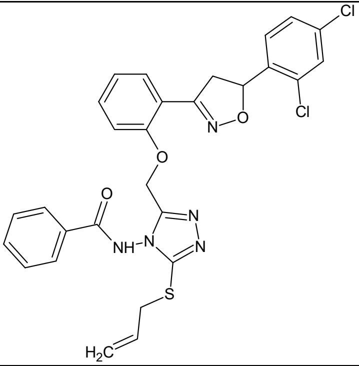	PDS155	580.484	128	3.656
19.	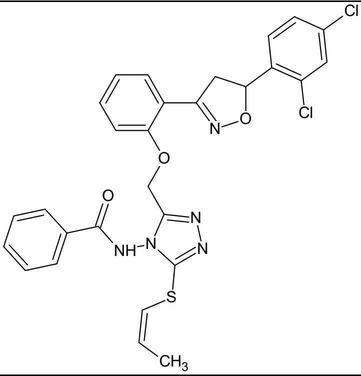	PDS156	580.484	128	3.656

20.	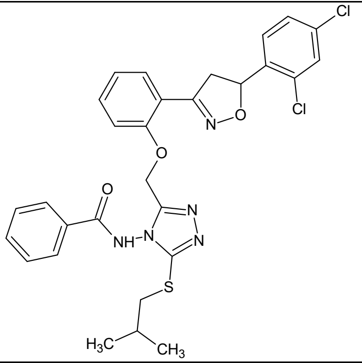	PDS157	596.527	512	3.066
21.	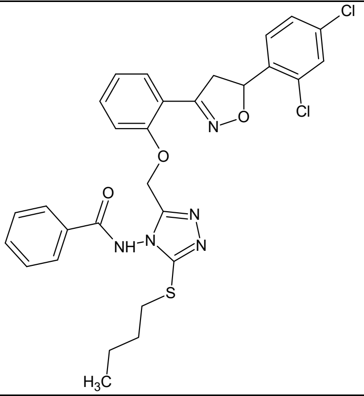	PDS158	596.527	64	3.969
22.	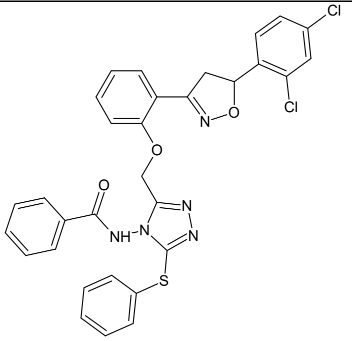	PDS159	616.517	256	3.381
23.	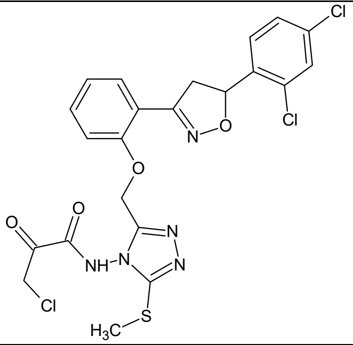	PDS160	554.833	32	4.239

24.	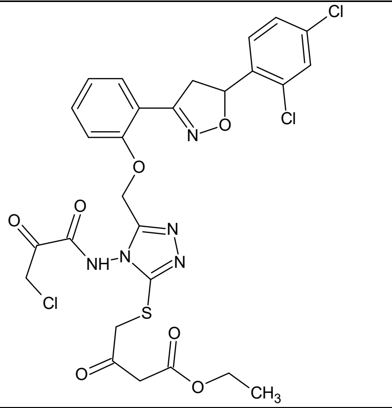	PDS161	668.932	128	3.718
25.	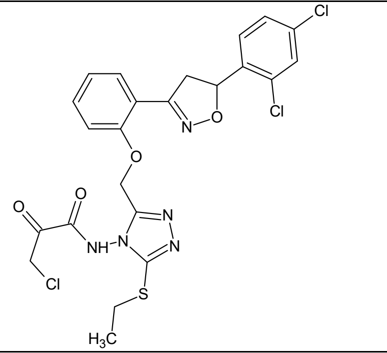	PDS162	568.86	512	3.045
26.	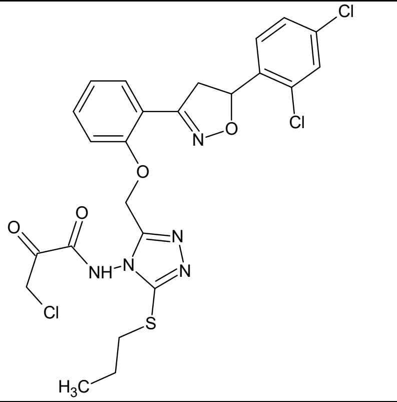	PDS163	582.886	4	5.163
27.	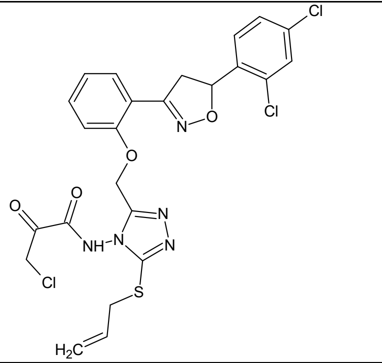	PDS164	580.870	256	3.355

28.	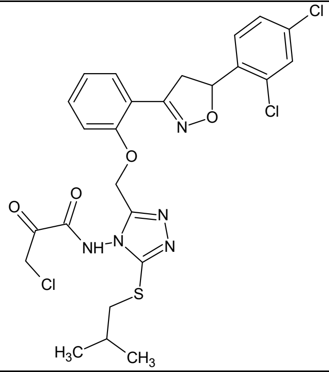	PDS166	596.913	1024	2.765
29.	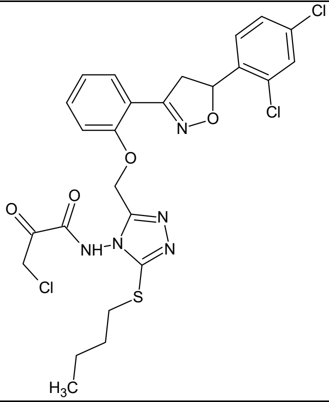	PDS167	596.913	32	4.270
30.	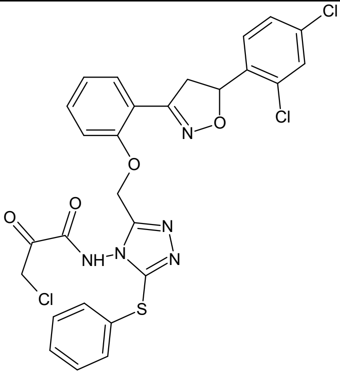	PDS168	616.902	128	3.683
