# Supplementary material for: Repurposing Based Identification of Novel Inhibitors against MmpS5-MmpL5 Efflux Pump of Mycobacterium smegmatis: A Combined In Silico and In Vitro Study
Source: Biomedicines. 2022 Jan 31;10(2):333. doi: 10.3390/biomedicines10020333 (PMC8869396; doi:10.3390/biomedicines10020333)
Supplement: Supplementary file 1 [file biomedicines-10-00333-s001.zip › Table S2.pdf]

Table S2: The list of assessment parameters generated for the developed QSAR models

S. No	QSAR model	R^2	Q^2	S.D.
1.	1	0.7499	0.8438	0.4268
2.	2	0.7697	0.8208	0.4081
3.	3	0.7704	0.8415	0.4251
4.	4	0.7784	0.8447	0.4306
5.	5	0.8946	0.8430	0.2881
6.	6	0.8734	0.8439	0.3254
7.	7	0.7502	0.8384	0.4449
8.	8	0.7491	0.8658	0.4260
9.	9	0.7648	0.8690	0.4303
10.	10	0.7797	0.7195	0.4293
