# Supplementary material for: Repurposing Based Identification of Novel Inhibitors against MmpS5-MmpL5 Efflux Pump of Mycobacterium smegmatis: A Combined In Silico and In Vitro Study
Source: Biomedicines. 2022 Jan 31;10(2):333. doi: 10.3390/biomedicines10020333 (PMC8869396; doi:10.3390/biomedicines10020333)
Supplement: Supplementary file 1 [file biomedicines-10-00333-s001.zip › Table S3.pdf]

Table S3: The comparison of the experimental and predicted pMICs on the basis of QSAR model 6

S. No	Inhibitor ID	Set	pMIC (Experimental)	pMIC (Predicted)	Error
1.	PDS168	Test	3.683	3.4599	-0.2231
2.	PDS167	Test	4.27	4.2647	-0.0053
3.	PDS166	Training	2.765	3.0185	0.2535
4.	PDS164	Test	3.355	3.2598	-0.0952
5.	PDS163	Training	5.163	5.086	-0.077
6.	PDS162	Training	3.045	4.0216	0.9766
7.	PDS161	Training	3.718	3.2516	-0.4664
8.	PDS160	Training	4.239	4.1495	-0.0895
9.	PDS159	Training	3.381	3.5788	0.1978
10.	PDS158	Training	3.969	4.3848	0.4158
11.	PDS157	Test	3.066	3.2951	0.2291
12.	PDS156	Training	3.656	3.3532	-0.3028
13.	PDS155	Training	3.656	3.4947	-0.1613
14.	PDS154	Test	5.464	5.1779	-0.2861
15.	PDS153	Training	5.152	4.955	-0.197
16.	PDS152	Training	3.115	3.0675	-0.0475
17.	PDS151	Training	4.238	4.2115	-0.0265
18.	PDS149	Training	4.222	4.6848	0.4628
19.	PDS147	Training	3.005	2.9609	-0.0441
20.	PDS146	Training	3.306	3.2262	-0.0798
21.	PDS145	Training	5.716	5.5139	-0.2021
22.	PDS144	Training	5.403	5.0594	-0.3436
23.	PDS143	Training	3.073	3.0562	-0.0168
24.	PDS142	Training	4.187	4.0483	-0.1387
25.	PDS141	Training	3.689	3.5451	-0.1439
26.	PDS140	Test	3.99	3.6456	-0.3445
27.	PDS138	Training	4.528	4.6926	0.1646
28.	PDS137	Test	4.187	4.6933	0.5063
29.	PDS136	Training	4.776	4.8494	0.0734
30.	PDS121	Training	5.338	5.1306	-0.2074
